# Recent Technical Advances in Sample Preparation for Single-Particle Cryo-EM

**DOI:** 10.3389/fmolb.2022.892459

**Published:** 2022-06-24

**Authors:** Yixin Xu, Shangyu Dang

**Affiliations:** ^1^ Division of Life Science, The Hong Kong University of Science and Technology, Kowloon, Hong Kong SAR, China; ^2^ Southern Marine Science and Engineering Guangdong Laboratory (Guangzhou), Guangzhou, China; ^3^ Center of Systems Biology and Human Health, The Hong Kong University of Science and Technology, Kowloon, Hong Kong SAR, China

**Keywords:** single-particle cryo-electron microscopy, sample preparation, air-water interface, grid modification, particle distribution

## Abstract

Cryo-sample preparation is a vital step in the process of obtaining high-resolution structures of macromolecules by using the single-particle cryo–electron microscopy (cryo-EM) method; however, cryo-sample preparation is commonly hampered by high uncertainty and low reproducibility. Specifically, the existence of air-water interfaces during the sample vitrification process could cause protein denaturation and aggregation, complex disassembly, adoption of preferred orientations, and other serious problems affecting the protein particles, thereby making it challenging to pursue high-resolution 3D reconstruction. Therefore, sample preparation has emerged as a critical research topic, and several new methods for application at various preparation stages have been proposed to overcome the aforementioned hurdles. Here, we summarize the methods developed for enhancing the quality of cryo-samples at distinct stages of sample preparation, and we offer insights for developing future strategies based on diverse viewpoints. We anticipate that cryo-sample preparation will no longer be a limiting step in the single-particle cryo-EM field as increasing numbers of methods are developed in the near future, which will ultimately benefit the entire research community.

## Introduction

**FIGURE 1:**
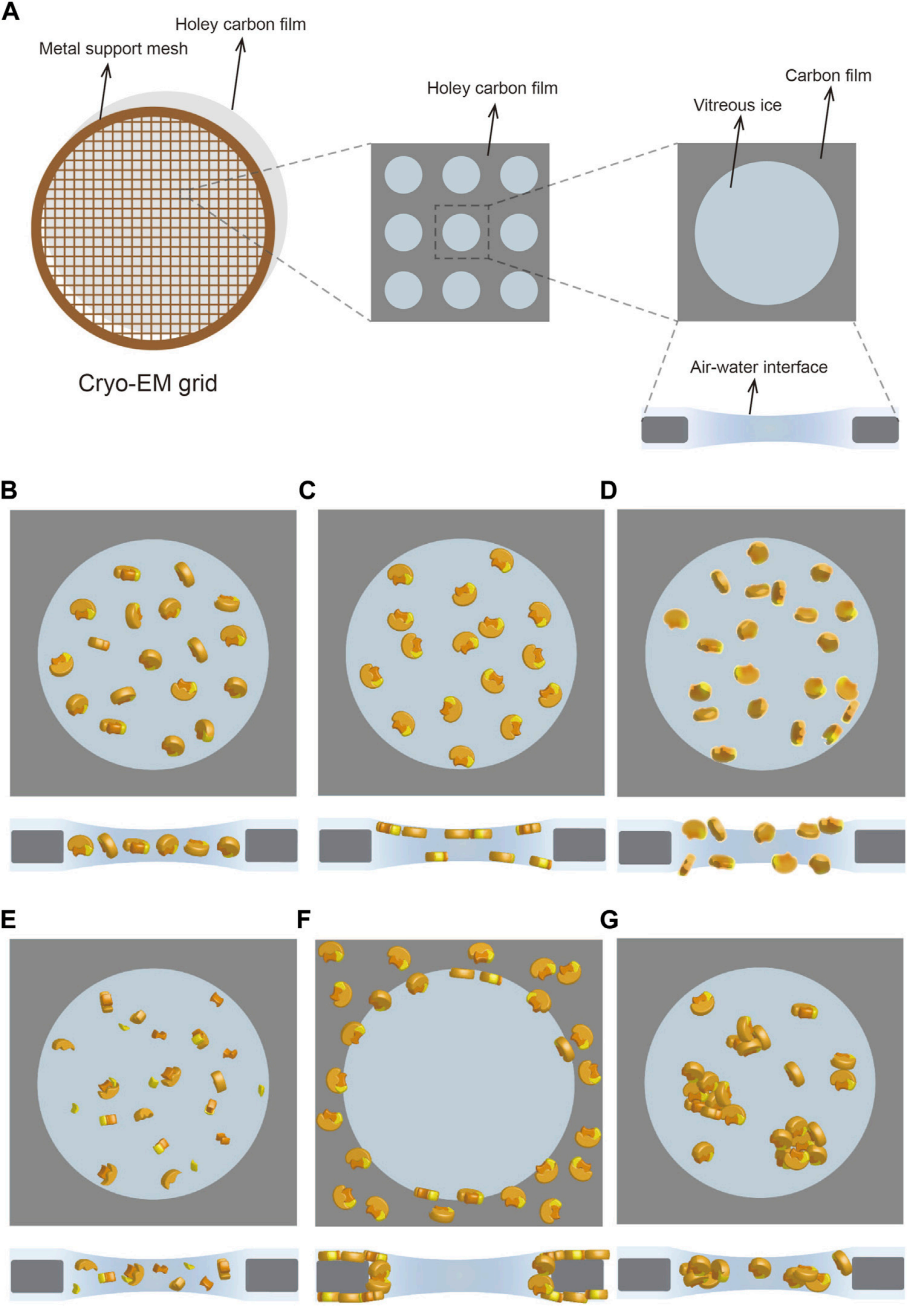
Cryo-specimen in single-particle cryo-EM. **(A)** Cryo-EM grid usually has a metal support mesh and a holey carbon film to carry the sample solution. The particles are frozen in vitreous ice in the holes for image acquisition **(B–G)** Different states of particles in vitreous ice, including ideal situation **(B)**, preferred orientation **(C)**, denaturation **(D)**, disassembly **(E)**, adsorption on carbon film **(F)**, and aggregation **(G)**. Subunits in the protein complex are shown in yellow, orange, and lemon, respectively.

Single-particle cryo-electron microscopy (cryo-EM) is emerging as one of the most effective techniques in the field of structural biology (Lyumkis, 2019; Bai, 2021). Purified biological macromolecules and/or complexes, isolated from endogenous or recombinant overexpression sources, are first applied to cryo-EM grids and rapidly frozen to generate an extremely thin layer of vitreous ice (Figure 1A). Next, the cryo-specimen is transferred to an electron microscope, and the images are acquired at liquid nitrogen temperature. Lastly, single particles are boxed out for iterative classification to sort out suitable particles for the final three-dimensional (3D) reconstruction (Cheng et al., 2015; Lyumkis, 2019). The obtained structural details of biological macromolecules at atomic resolution provide valuable information for not only unravelling the fundamental mechanisms of myriad biological processes but also driving the development of drugs for treating diseases caused by the dysfunctional biological macromolecules (Zhu et al., 2018; Han et al., 2022; Liang et al., 2022). Recent technical breakthroughs in single-particle cryo-EM have created a “resolution revolution” in structural biology by circumventing the major challenges faced when using the traditional X-ray crystallography methods (Cheng, 2015; Cheng, 2018).

The technical breakthroughs in cryo-EM, such as advances in hardware and updating of software, are attracting increasing attention in the field of structural biology (Egelman, 2016). The key driver of this “resolution revolution” is the replacement of the traditional charge-coupled device (CCD) cameras with new direct detection device (DDD) cameras (McMullan et al., 2016; Hurtley, 2018). The DDD camera can detect electrons directly at higher frame rates than the CCD camera, which results in relatively higher detective quantum efficiency and enables movie recording during exposure to correct the beam-induced image blurring (Li et al., 2013; Bai et al., 2015; McMullan et al., 2016). Moreover, introduction of new data processing algorithms, such as RELION (Scheres, 2012), Frealign (Grigorieff, 2016), cisTEM (Grant et al., 2018), cryoSPARC (Punjani et al., 2017), and automation of the work pipeline (Wang et al., 2016; Li et al., 2020; Stabrin et al., 2020) have also led cryo-EM technology into a new era. In 2020, cryo-EM hardware improvements pushed resolution to the atomic level (Nakane et al., 2020; Yip et al., 2020; Zhang et al., 2020), refreshing a limitation of cryo-EM structures. By contrast, the development of sample preparation methods in cryo-EM has been slowed by several obstacles (Passmore and Russo, 2016), and this has widely hindered the structure determination studies in the field.

Sample preparation in cryo-EM generally consists of two key procedures: purification and vitrification (Weissenberger et al., 2021). The first step in the macromolecular structure analysis is the preparation of purified macromolecules in an optimal biochemical environment (Passmore and Russo, 2016). However, obtaining a high-quality specimen after purification does not guarantee a suitable cryo-specimen after vitrification. This is because cryo-grid preparation is a trial and error process that is affected by diverse factors such as grid material, glow discharge, incubation, and blotting (Weissenberger et al., 2021). Variations of these external factors and the characteristics of the samples themselves could lead to an undesirable state of the particles, making it challenging to reconstruct a high-resolution structure. In real-world cases, the adoption of all preferred orientations by particles and the denaturation, disassembly, adsorption on carbon film, and aggregation of particles (Figures 1B–G) are problems commonly encountered in sample preparation (Drulyte et al., 2018). Besides sample quality at the biochemical level, the main reason for these recurring problems is the adsorption of particles on the air–water interface (AWI) during vitrification (Glaeser and Han, 2017; Glaeser, 2018).

The AWI can severely affect particles because the extremely thin layer of liquid remaining on the cryo-grid after blotting can generate a large surface-to-volume ratio (Glaeser and Han, 2017). Typically, free-floating particles collide with the AWI thousands of times before vitrification (Klebl et al., 2020) even though plunge freezing takes only a few seconds. When the particles reach the AWI, certain hydrophobic side chains or small hydrophobic patches on the surface can facilitate particle adsorption on the AWI. Whereas slight adsorption might generate preferred orientations, substantial adsorption could result in partial or complete denaturation due to hydrophobic residues being exposed to air but hydrophilic residues being retained in water (Glaeser and Han, 2017; Glaeser, 2018). Although this protein-unfolding interaction at the AWI occurs within a very short time, the interaction can lethally affect subsequent structural analysis (Klebl et al., 2020). Thus, diverse methods have been proposed to reduce the effect of the AWI (Tan et al., 2017; Noble et al., 2018; Chen et al., 2019; Wang and Yu, 2020), but the AWI influence remains a challenging problem to solve comprehensively.

Here, we summarize and classify distinct strategies aimed at improving the quality of cryo-specimens. We illustrate the technical methods and list the benefits and drawbacks of these strategies to compare the differences between the strategies and develop an evaluation system. We also offer certain new perspectives for optimizing the particle behavior in vitreous ice in order to facilitate high-resolution structure determination by using single-particle cryo-EM.

## Sample Biochemistry

Purification of an adequate amount of high-quality protein samples is the key for high-resolution cryo-EM studies. Besides the technique used to isolate the target protein at a high level of purity, the buffer condition is another critical factor because it not only helps ensure a well-behaved protein in solution but also affects protein behavior in vitreous ice when preparing the cryo-specimen (Cheung et al., 2013; Glaeser et al., 2016; Passmore and Russo, 2016). Accordingly, the same proteins purified under distinct conditions display divergent behaviors in vitreous ice (Dang et al., 2017; Paulino et al., 2017; Feng et al., 2019). Therefore, screening of the purification conditions, including but not limited to pH, salt, ligands, small molecules, and detergents (for membrane proteins), relies heavily on the properties of the target protein itself, and this represents one approach for improving the cryo-sample quality. The efficiency of the screening purification conditions, which can be a time-consuming process, could be increased by introducing several screening and optimization methods to overcome the compositional heterogeneity problems of the protein sample (Table 1). The thermofluor screening assay (Ericsson et al., 2006), which is typically used to identify the appropriate buffer conditions for protein stabilization and crystallization, reports the melting temperature of a protein based on fluorescence and thereby reveals the distinct states of the sample, such as protein denaturation and disassembly. ProteoPlex (Chari et al., 2015), CPM (Alexandrov et al., 2008; Ashok and Jaakola, 2016), DSF-GFP (Moreau et al., 2012), and MoltenProt (Kotov et al., 2021) can serve the same purpose as thermofluor screening to enhance the sample quality by monitoring the unfolding behavior of a protein. Furthermore, methods have also been developed to facilitate sample optimization, as exemplified by mass photometry (MP) (Sonn-Segev et al., 2020; Olerinyova et al., 2021), size-exclusion chromatography coupled to multi-angle light scattering (SEC-MALS) (Some et al., 2019), and other similar methods.

**TABLE 1:** Summary of biochemical methods to improve the quality of cryo-specimen.

	Method	Mechanism	Representative research
Condition Screening	Thermal unfolding screening	Improvement of buffer optimization by screening thermal unfolding behavior of proteins	**ThermoFluor**: Assess thermal stability of protein under varying conditions by comparing its melting points in a thermofluor-based high-throughput approach (Ericsson et al., 2006). **DSF-GFP**: Assess GFP-tagged protein stability by using differential scanning fluorimetry (DSF) in a high-throughput way (Moreau et al., 2012). **ProteoPlex**: Optimize stability, homogeneity, and solubility of protein complexes by screening thermal unfolding behavior under variant buffer conditions (Chari et al., 2015); cryo-EM structure of HTT-HAP40 complex at 4 Å (Guo et al., 2018). **CPM**: Assess membrane protein stability by using thiol-specific fluorochrome N-[4-(7-diethylamino-4-methyl-3-coumarinyl)phenyl]maleimide (CPM) (Alexandrov et al., 2008; Ashok and Jaakola, 2016)
	Mass photometry	The light scattering of single molecules is quantified to determine the molecular mass	Evaluate sample homogeneity and complex stability by measuring the molecular mass of the sample (Sonn-Segev et al., 2020; Olerinyova et al., 2021); structural studies of mutant ACE2 and RBD complex (Higuchi et al., 2021)
	SEC-MALS	MALS combined with SEC to characterize sample behavior by measuring molecular mass with a higher accuracy	Facilitate cryo-EM structure determination of NLRP3 at 3.9 Å (Hochheiser et al., 2022)
Stabilization of complex	Chemical crosslinking	Inter cross-links make protein stable	Crosslinking to stabilize complex for structure studies of rhodopsin dimers at 4.7 Å (Zhao et al., 2019) and Drosha-DGCR8–pri-miRNA complex at 3.7 Å (Partin et al., 2020) by using single-particle cryo-EM
	GraFix	Combination of glycerol gradient centrifugation and crosslinking to prevent denaturation and aggregation	GraFix to stabilize complex for cryo-EM studies of MCM double hexamer bound with Dbf4-Cdc7 kinase (Cheng et al., 2022), human spliceosome at 3.7 Å (Zhang et al., 2017), preinitiation complex (Chen et al., 2021), and ALB1 nucleosome at 4.0 Å (Takizawa et al., 2018)
	Crosslinking coupled SEC	SEC combined with crosslinking to increase homogeneity of the sample	Stabilize complex for structural study of β_2_V_2_R-β-arrestin-1-Fab30 (28.8 Å) (Shukla et al., 2014), RNA polymerase I-Rrn3 (4.8 Å) (Engel et al., 2016), *E. coli* RNAP TEC (4.1 Å), and Nun/TEC (3.07 Å) complexes (Kang et al., 2017)
	AgarFix	Prevent aggregate formation during chemical crosslinking by incorporating the agarose matrix	Stabilize complex of Spt-Ada-Gcn5 acetyltransferase (SAGA) and prevent denaturation and aggregation (Adamus et al., 2019)
	GraDeR	Glycerol gradient centrifugation can remove free detergent monomers and micelles	GraDeR method used to remove extra detergent in sample for structural studies of monomeric PSI at 3.2 Å (Çoruh et al., 2021) and innexin-6 gap junction channel at 3.6 Å (Oshima et al., 2016)
Additives	Detergents	Detergent molecules occupy and change feature of AWI	Adding CHAPSO to remove orientation bias for RNA polymerase at 3.5–4 Å (Chen et al., 2019). Adding CATB to change particles’ orientation distribution for HA trimer at 3.3 Å (Li et al., 2021)
Protein modification	Protein PEGylation	Change surface charge of proteins by modifying primary amines with PEG chain	Cryo-EM studies of PEGylation-modified β-amylase at 2.3 Å, ADH at 3.3 Å, and NOD2 at 3.7 Å (Zhang et al., 2021)

Macromolecular complex disassembly is another recurrent problem in cryo-sample preparation, particularly in the case of a complex in which the inter-subunit interactions are weak and/or transient. Chemical crosslinking has been used to stabilize protein complex for structural studies (Zhao et al., 2019; Partin et al., 2020), despite that the heterogeneity (subcomplex, aggregation, and unspecific artifacts) could be introduced by direct crosslinking in many cases. GraFix, a combination of gradient centrifugation and crosslinking, can be used to stabilize the protein complexes in a highly homogeneous state suitable for cryo-EM studies by removing the aggregation and undesired subcomplexes (Kastner et al., 2008). Similarly, combinations of chemical crosslinking with other approaches, as with size-exclusion chromatography (Shukla et al., 2014) and AgarFix (an agarose matrix–based method) (Adamus et al., 2019), provide an alternative strategy for improving the cryo-sample quality in the case of large complexes. For membrane proteins and protein complexes, GraDeR, a strategy based on glycerol gradient centrifugation, has been shown to enhance sample homogeneity by efficiently and gently removing excess detergents and micelles (Hauer et al., 2015).

Two other common strategies employed at the sample biochemistry state to improve cryo-sample behavior are the alteration of the solution environment by including additives (Chen et al., 2019; Li et al., 2021) and modification of the target protein itself (Zhang et al., 2021).

In certain cases, adding specific detergents at a low concentration can reduce the particle adsorption on the AWI. As amphipathic molecules, detergents are commonly used during the purification of membrane proteins to protect and stabilize the exposed hydrophobic regions of the proteins by mimicking the environment of the lipid bilayer (Autzen et al., 2019). Moreover, a few detergents, such as CHAPSO, can enhance the quality of cryo-specimens, when included as extra additives. CHAPSO, a zwitterionic detergent, was shown to mitigate the preferred orientation problem in the case of bacterial RNA polymerase (Chen et al., 2019). Moreover, different types of detergents distinctly affect the interactions between proteins and the AWI: whereas cationic and anionic detergents exert little effect, certain nonionic and zwitterionic detergents can keep the protein particles away from the AWI (Li et al., 2021). The potential underlying mechanism here is that nonionic and zwitterionic detergents (compared to other detergents) occupy the AWI without protein adsorption due to their neutral charge properties, and thus yield more evenly distributed particles in vitreous ice. Intriguingly, adding a very low concentration (0.002–0.005%) of cetyltrimethylammonium bromide (CTAB), a cationic detergent, enabled the high-resolution structure determination of a hemagglutinin (HA) trimer by introducing particles those exhibit side-view features, although the protein particles remained attached to the AWI (Li et al., 2021).

Modification of protein properties, particularly surface properties, might alter protein behavior in vitreous ice. A PEGylation method has been reported to reduce the AWI influence by modifying the exposed primary amines in proteins (Zhang et al., 2021). The PEG chains attached in the PEGylated proteins could form a shielding layer on the surface to keep the protein particles away from the AWI and improve particle behavior in vitreous ice as compared to the behavior of unmodified proteins. Notably, the structures of PEGylated proteins are almost identical to those of their unmodified counterparts. This suggests that PEGylation is a mild modification that barely introduces any artificial features in the structure determination process, as demonstrated in the case of several proteins, such as apoferritin and β-galactosidase (Zhang et al., 2021).

Introducing additives, such as detergents, in the preparation of cryo-specimens is a simple process, much like the conventional protocols. However, our practical experience in cryo-EM sample preparation indicates that distinct proteins respond differently to the inclusion of additives. Thus, identification of potential additives through screening might consume a considerable amount of effort and time, particularly if data collection is required to evaluate the effect of the additives. Furthermore, in the case of several samples, identifying suitable additives and optimal conditions might be extremely challenging, if not impossible. Modification of protein properties, as exemplified by PEGylation, is also a protein-dependent approach, and although a modified protein shares identical structures with the unmodified protein, the possibility that these chemical modifications could generate artificial structures cannot be excluded. Nevertheless, these optimization methods at the protein sample level have provided approaches to enhance the quality of cryo-specimens and are thus suitable for application during sample preparation, particularly considering that the methods involve simple processes.

## Grid Fabrication

Sample solutions must be loaded onto the cryo-grids for vitrification before being transferred to an electron microscope for image acquisition. Cryo-EM grids typically include a metal (copper, gold, nickel, and others) support bearing different amounts of mesh mounted with a holey carbon film featuring distinct patterns of holes to satisfy the requirements of distinct samples (Figure 1A). Replacement of the holey carbon film by a highly conductive holey metal film, such as a film of gold (Russo and Passmore, 2014) or amorphous nickel–titanium alloy (ANTA) (Huang et al., 2020), can improve the image quality by reducing beam-induced motion. Moreover, hydrophilic treatment of the cryo-grids through glow discharging or plasma cleaning is critical for carrying the sample solution because the carbon film is a hydrophobic substance (Passmore and Russo, 2016). In the case of samples that are prone to being adsorbed on the carbon film, this can make high-resolution reconstruction challenging due to the collection of an insufficient number of particles. The reasons for protein particles failing to enter holes can be complex, and optimizing the sample itself, such as by increasing the protein concentration, might help in certain cases. Another promising strategy is the modification of cryo-grids by introducing carbon film-like properties in the holes (Table 2). Coating the holey carbon grids with a thin layer (2–4 nm) of continuous carbon to eliminate the influence of this adverse adsorption has long been used as a strategy in cryo-EM studies of the ribosome (Grassucci et al., 2007; Bai et al., 2013) and in other cases (Nguyen et al., 2018; Liu et al., 2020; Perederina et al., 2020). Although application of a continuous carbon layer in cryo-grids has enabled the determination of high-resolution structures of several proteins, the thin layer of carbon reduces the signal-to-noise ratio (SNR) of micrographs, leading to a further loss of high-resolution information, particularly for small proteins.

**TABLE 2:** Summary of methods in grid fabrication, device development, and data collection to improve the quality of cryo-specimen.

	Method	Mechanism	Representative Research
Grids fabrication	Carbon film grids	Continuous carbon film attracts proteins	Apply on 80S ribosome at 4.5 Å (Bai et al., 2013), lipid transporter YebT at 3.0 Å (Liu et al., 2020), and RNase MRP holoenzyme at 3.0 Å (Perederina et al., 2020)
	Graphene and graphene oxide grids	Graphene and graphene oxide attract proteins into holes	Graphene grids: streptavidin at 2.6 Å (Han et al., 2020), AcrB embedded in liposomes at 3.9 Å (Yao et al., 2020), and 20S proteasomes at 2.36 Å (Zheng et al., 2020); Graphene Oxide grids: 20S proteasome at 2.5 Å (Palovcak et al., 2018) and separase–securing at 3.8 Å (Boland et al., 2017)
	Affinity tag grids	Chemical groups linked with grid surface can capture proteins	Graphene oxide based grids: TRPA1 at 3.5 Å (Wang and Yu, 2020) and 3.3 Å (Wang and Liu, 2020); Lipid monolayer based grids of 50S ribosomal subunit at 21 Å, 30S ribosomal subunit at 24 Å, 70S ribosome at 28 Å, (Kelly et al., 2008) and RNA polymerase II at 25 Å (Kelly et al., 2010); Streptavidin crystal film based grids of 70S ribosomes at 4.0 Å (Han et al., 2016)
	HFBI film grid	Hydrophilic side of the HFBI film can adsorb proteins	HFBI grids of haemoglobin at 3.60 Å, aldolase at 3.28 Å, HA trimer at 2.56 Å, catalase at 2.29 Å, GDH at 2.26 Å, and apoferritin at 1.96 Å (Fan et al., 2021)
Sample preparation device	Fast vitrification	Reduce dwell time to avoid particles adsorbed to AWI by spraying or printing sample to grids using different ways	Apply Spotiton on HA trimer at 3.77 Å and insulin receptor at 4.93 Å (Noble et al., 2018) and 70S ribosome at 4.75 Å (Dandey et al., 2020); Apply microfluidic device on apoferritin at 2.77 Å (Mäeots et al., 2020) and 3.0 Å (Feng et al., 2017); apply VitroJet on apoferritin at 2.49 Å, GroEL at 2.94 Å, worm hemoglobin at 3.11 Å, and beta-galactosidase at 3.11 Å (Ravelli et al., 2020); apply ultrasonic spray device on 70S ribosome at 3.4 Å (Ashtiani et al., 2018) and apoferritin at 2.6 Å (Rubinstein et al., 2019); apply cryoWriter on urease at 5.03 Å (Arnold et al., 2017)
	Nanofluidic chip	Electron-transparent nanochannels encapsulate sample solution	Apply nanofluidic chip on apoferritin at 2.99 Å, TMV at 3.65 Å, and T20S at 5.42 Å (Huber et al., 2022)
Data collection	Tilting	Obtain more views of particles by collecting data at tilted angles	Addressing the preferred orientation of HA trimer at 4.2 Å (Tan et al., 2017)

Introduction of graphene and graphene derivatives to modify cryo-grids can address the problem of SNR reduction and is becoming a mainstream strategy in cryo-grid modification (Table 2) (Palovcak et al., 2018; Han et al., 2020; Wang and Liu, 2020; Wang and Yu, 2020). Attachment of a continuous monolayer of graphene, invisible in the high-resolution range of cryo-EM due to its conductive property (Naydenova et al., 2019), to a holey carbon foil markedly increased the amount of particles in holes. Furthermore, structure determination of a 52-kDa streptavidin at 2.6 Å also confirmed the suitability of graphene grids for high-resolution cryo-EM analysis of small proteins (Han et al., 2020). As compared with graphene, graphene oxide (GO) is more widely used because of its hydrophilic property and capability of introducing other functional groups, such as covalently linked affinity tags. Such GO-based affinity grids can improve particle distribution and orientation in vitreous ice by keeping the particles away from the AWI through interactions with chemical groups covalently linked to the GO layer (Wang and Liu, 2020; Wang and Yu, 2020). Monolayer graphene grids can also be covalently functionalized by using a plasma surface-modification system with different chemicals, as in the case of the amylamine-functionalized graphene grid (Naydenova et al., 2019).

**FIGURE 2:**
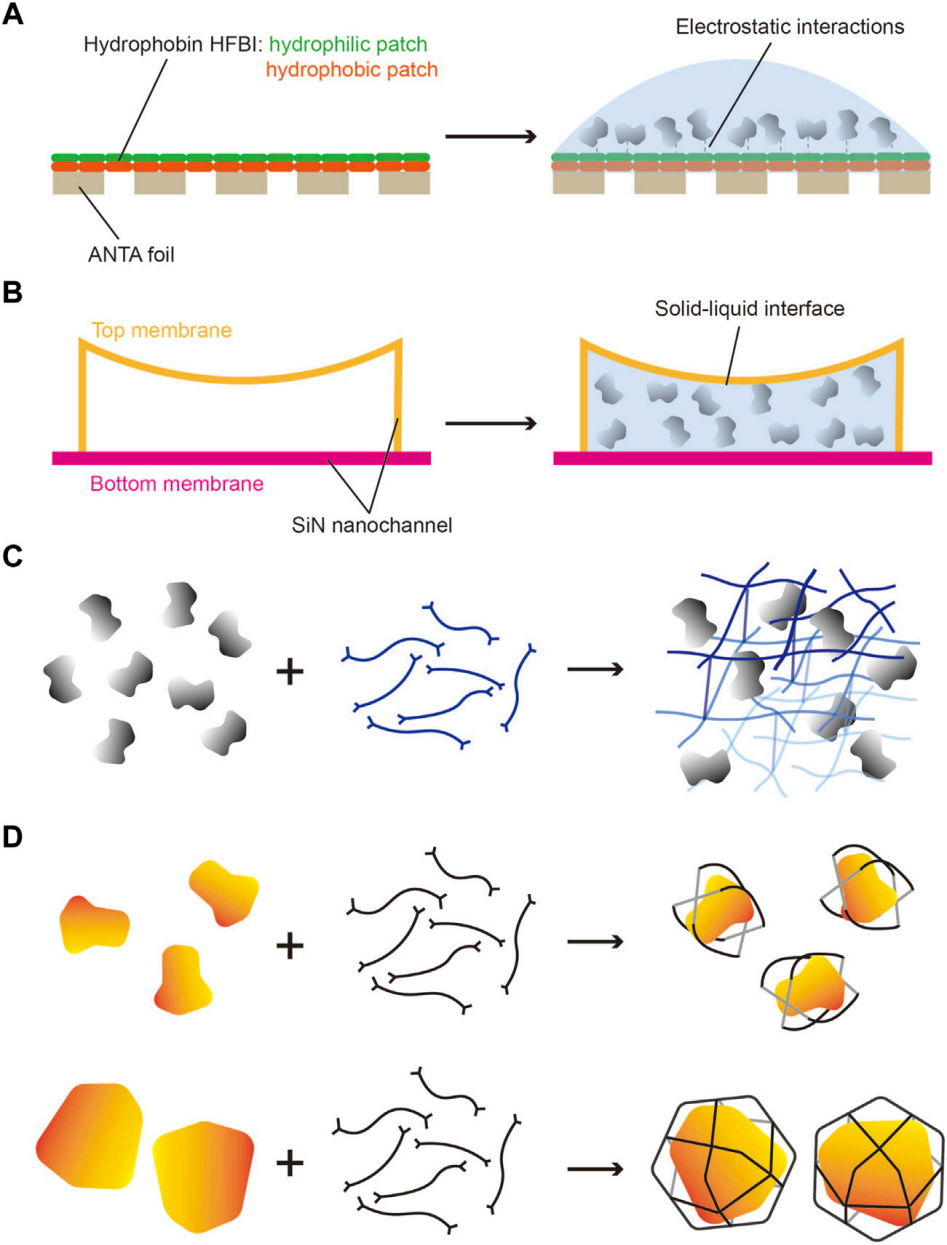
Potential strategies for addressing problems in cryo-sample preparation. **(A)** HFBI grid has a continuous hydrophobin HFBI 2D crystals film on the ANTA foil. The hydrophilic patches of HFBI load sample and adsorb particles by electrostatic interactions to keep particles away from the AWI. **(B)** Nanofluidic cryo-chip has many electron-transparent nanochannels made of SiN_X_ membrane. The sample solution is applied in closed nanochannels by capillary action without the AWI in vitrification. **(C)** 3D chemical network can be designed to fix particles in different orientations and uniform distribution. **(D)** Some chemical cages can be used to carry protein particles of different sizes to protect particles away from the AWI and provide random orientations.

Other cryo-EM grids modified to serve as the affinity substrate include grids coated with lipid monolayers containing Ni-NTA–functionalized lipids (Kelly et al., 2008; Kelly et al., 2010) and 2D streptavidin crystal films (Wang et al., 2008; Han et al., 2016) (Table 2). Recently, a new cryo-grid featuring a support film formed by the 2D crystals of hydrophobin HFBI based on the ANTA foil was developed to facilitate high-quality cryo-sample preparation (Table 2) (Fan et al., 2021). The amphipathic HFBIs adhere to the AWI and form a crystalline monolayer film through self-assembly, and this effectively sequesters the particles away from the AWI (Figure 2A) and thereby eliminates the preferred orientation problem and enables high-resolution structural analysis of small proteins (Fan et al., 2021).

Grids coated with graphene or graphene oxide are applicable to a wide range of macromolecules (Boland et al., 2017; Narayanan et al., 2018; Rapisarda et al., 2019; Yao et al., 2020) with which problems might be encountered in vitrification. The procedure of cryo-sample preparation employing graphene and GO grids as the carrier is as simple as the procedure involving the use of conventional holey grids. Although the fabrication process required for the new method is becoming progressively easier and more widely available than before, additional materials and specific operations are required for the preparation of GO grids (Palovcak et al., 2018; Han et al., 2020). Moreover, variations in the quality of commercially available graphene and GO grids lower the efficiency of grid preparation, and surface contamination of these grids also affects protein behavior after vitrification (Palovcak et al., 2018; Lin et al., 2019). The emergence of HFBI girds points to a new direction in grid modification, although practical application here requires further investigation. Functionalized graphene membrane (FGM) grids have also been reported, wherein the bioactive ligands are linked on the monolayer graphene, and these exhibit affinity for His-tagged proteins and show improved particle distribution (Liu et al., 2019). However, the application of affinity grids currently remains limited due to the requirement of specific binding with a target protein. Although future improvements are necessary, the incorporation of protein purification into cryo-sample preparation by using the affinity grids warrants attention because this can considerably lower the complexity of sample preparation and reduce the time cost. Specifically, affinity grids developed based on graphene and GO films are expected to attract an increased interest in the future (Liu et al., 2019; Wang and Liu, 2020). Realization of grid fabrication and high specificity represent two factors for future improvement in the preparation of affinity grids, and the shelf-life of the affinity grids must also be considered in the optimization process.

## Sample Preparation Device

The most widely used cryo-EM sample preparation devices for plunge freezing include Vitrobot (Thermo Fisher Scientific), Cryoplunge 3 (Gatan), and EM GP (Leica) (Table 3). The cryo-specimen are prepared by pipetting a few microliters of the sample solution on a cryo-grid and incubating for several seconds (or not incubating for an unsupported foil grid) to facilitate particle deposition. Subsequently, the grid is blotted with a filter paper to remove excess solution and then plunge-frozen in liquid ethane cooled by liquid nitrogen. With the use of these conventional devices, the dwell time of the specimen on the grids before freezing is at the level of seconds, but this dwell time is adequately long for specimen particles to be adsorbed on the AWI. In order to avoid the AWI interference, various devices have been designed to reduce the time of vitrification to the millisecond level through rapid freezing without blotting, including devices employing inkjet printing (Spotiton) (Jain et al., 2012; Noble et al., 2018; Dandey et al., 2020), gas pressure spray (microfluidic devices) (Feng et al., 2017; Kaledhonkar et al., 2018; Mäeots et al., 2020), ultrasonic spray (Ashtiani et al., 2018; Rubinstein et al., 2019), and electrostatic spray (White et al., 2003). Here, we mainly focus on Spotiton and the microfluidic devices (Table 2).

**TABLE 3:** Comparison of widely used devices for plunge freezing.

Features	Vitrobot (Thermo Fisher Scientific)	Cryoplunge 3 (Gatan)	EM GP (Leica)
Automaticity	Automatic	Semiautomatic	Automatic
Humidity	Ambient—100%	Ambient—100%	Ambient—99%
Working temperature	4–60°C	4—Ambient	4–60°C
Blot manner	Two side	One or two side	One side +/− the other side
Ice shape (blot angle)	Angular	Straight	Straight
Cryogen temperature monitor	No	Yes	Yes
Multiple loading	No	Yes	Yes
Multiple blotting	Yes	Yes	Yes
Foot pedal	Yes	No	Yes
Blotting force	Yes	Yes (manual)	Yes
Pre-blotting time	Yes	Manual	Yes
Drain time	Yes	No	Yes
Moving apparatus	Ethane container	Safety shield	Chamber
Stereomicroscope	No	No	Yes
LN2 overflow control	No	Yes	Yes
Filter paper	Automatic rotate	Manual rotate	Automatic rotate

In the Spotiton system, picoliter-sized droplets of the sample solution are sprayed on the grid by using the inkjet mechanism. The total volume (number of droplets) can be accurately dispensed through the piezoelectric inkjet head by applying voltage pulses to control the thickness of ice (Jain et al., 2012). Noble et al. showed that reducing the dwell time (spot-to-plunge time) on the grids to hundreds of milliseconds or even less when using the Spotiton system could substantially decrease the particles’ opportunities to be adsorbed on the AWI (Noble et al., 2018). An increase in the non-adsorbed particles in vitreous ice enhanced the particle distribution and alleviated the preferred orientation problem. Dandey *et al.* further pushed the limit of the dwell time to 50 ms for the Spotiton system and enabled time-resolved cryo-EM studies through the capture of intermediate states (Jain et al., 2012; Noble et al., 2018; Dandey et al., 2020). Based on this high-resolution and fast-freezing system, Spotiton has been commercialized in the form of a next-generation device, Chameleon (Darrow et al., 2019). Moreover, the “self-wicking” nanowire grids applied in combination with Spotiton can wick away excess liquid and generate an ice layer of suitable thickness for high-quality image acquisition (Wei et al., 2018).

A microfluidic device designed for time-resolved cryo-EM contains a microfluidic chip for the incubation of reactants, and a nozzle under gas pressure to spray the sample on the grid. The microfluidic time-resolved cryo-EM was used for demonstrating the dynamics of the ribosome complex in bacterial translation initiation, with the resolution at 3.9 Å for the 70S elongation-competent complex (Kaledhonkar et al., 2019). The time of incubation can be tuned from 10 to 1,000 ms to capture the distinct intermediate states by adjusting the length of the channel integrated on the chip (Feng et al., 2017). By using a microfluidic device, the dwell time (spray-to-plunge time) of the sample on the grids can be reduced to as little as 2.5 ms to further restrain particle adsorption on the AWI. A subsequent study further showed that a modular microfluidic system, featuring a dwell time as low as 16 ms and a minimal solution volume of ∼20 μl, improved the particle distribution of apoferritin and eliminated the aggregation of the CSN^5H138A^-SCF-N8^Skp2/Cks1^ complex in the grid hole (Mäeots et al., 2020). Recently, a nanofluidic chip has been reported in preparing the cryo-EM samples with increased reproducibility (Huber et al., 2022). The cryo-chip contains several nanofluidic channels formed by thin silicon-rich nitride (SiN_x_) membranes to provide a predefined space to control ice thickness while maintaining electron transparency for cryo-EM imaging (Figure 2B). Because the sample solution here is encapsulated in nanochannels by using a microelectromechanical system, the AWI is replaced by a solid–liquid interface, thus providing a new strategy to avoid the influence of the AWI.

Another advantage of the aforementioned printing and spraying sample preparation devices is that the devices can be used in time-resolved cryo-EM studies (Chen and Frank, 2016; Kontziampasis et al., 2019; Dandey et al., 2020; Mäeots et al., 2020). The reaction time can be limited to milliseconds to allow the capture of intermediate states and thus depict the transient biological reaction in high resolution. However, these methods require larger amounts of sample solution at higher concentration than in the case of the blotting method (Klebl et al., 2020), which is not suitable for proteins that cannot be readily purified in a large quantity. Moreover, the extremely short time used for sample incubation on the grid might result in the sample droplet not spreading sufficiently and the particles not being deposited completely on the grid surface. Consequently, generating high-quality ice for data collection is time consuming (Klebl et al., 2020). Furthermore, although the nanofluidic cryo-chip featuring the SiN_x_ membrane eliminates the AWI problem, the solid–liquid interfaces can also affect the distribution of particles (Huber et al., 2022).

Notably, the design of the nanofluidic system also represents an attempt to automate the cryo-EM workflow and facilitate the sample preparation process (Huber et al., 2022). Whereas automation of the cryo-EM data processing has been widely studied (Li et al., 2020; Maruthi et al., 2020; Stabrin et al., 2020), research on automation of the sample preparation process remains limited. If the distinct procedures involved can be integrated, the efficiency of sample preparation will be improved markedly. The VitroJet device was reported as an integrated system, which is composed of a glow-discharge module, a process chamber, a pin printing system, and a vitrification module. The total time is ∼3 min with this automation, which thus serves as a template for the other automation designs (Ravelli et al., 2020).

## Data Collection

In the case of samples that adopt preferred orientations and the problem cannot be alleviated by using the aforementioned strategies focused on the sample preparation stage, an alternative approach could be applied in data collection. To compensate for the information missing due to the adoption of the preferred orientations, the specimen is tilted during data collection. In practice, the smallest titling angle is selected because large tilting angles cause instability and difficulty in data collection and data processing, respectively. The data collected from the different tilting angles can provide additional views that are missed due to the preferred orientations and thus improve map quality for 3D reconstruction. Tan et al. demonstrated the feasibility of this strategy by determining the structure of HA trimer at near-atomic resolution with less stretching and superior features in the reconstructed map from the dataset of 40°-tilted images (Tan et al., 2017). The generality and practicability of this method were also confirmed in subsequent studies (Davis et al., 2016; Balchin et al., 2018; Scapin et al., 2018).

The tilting method does not alter the sample characteristics and thus represents a sample-independent approach. However, micrographs collected at the titling angle introduce difficulties at the data processing stage. It is crucial to appropriately estimate the focus gradient at distinct tilting angles and apply additional accurate defocusing for individual particles, because tilting can cause variations in defocusing in the same micrograph. The constant tilting of the grid stage could also lead to increased beam-induced motion and loss of high-resolution information (Lyumkis, 2019). Furthermore, ice thickness is also inevitably increased, which lowers image contrast. Although data collection by using the tilting strategy can only solve a specific preferred orientation problem caused by the AWI, if superior algorithms improving the data processing of the tilted data emerge in the future, this method might be more widely applied (than currently) because of its sample-independent property.

## Discussion

Cryo-EM has become a mainstream method in structural biology for supporting numerous fundamental studies of various macromolecules. Moreover, in the majority of cases, cryo-EM now provides satisfactory results due to continuous improvements in hardware, such as the upgrading of direct electron detectors, and the application of Volta phase plate (Danev et al., 2014), chromatic aberration corrector (Cc) and other microscope accessories, and the cold field emission gun (CFEG) (Hamaguchi et al., 2019; Kato et al., 2019; Konings and Bischoff, 2020; Nakane et al., 2020), as well as software, such as for automated particle picking (Wang et al., 2016) and preprocessing (Maruthi et al., 2020). Conversely, the sample preparation process is the bottleneck that hinders high-resolution structure determination in several cases. To enhance the quality of cryo-specimens, different stages of cryo-sample preparation can be optimized.

Besides the classical techniques, new methods featuring creative strategies are emerging, such as those employing the HFBI grid (Fan et al., 2021) and the nanofluidic chip (Huber et al., 2022). In these new methods, special materials or unique designs are used to seek breakthroughs against the bottleneck of sample preparation, which is greatly encouraging for the cryo-EM community.

Recent work has also shown that the application of branched polymers in cryo-specimen preparation improves sample behavior in vitreous ice by keeping particles away from the AWI and thus protecting the particles (Xu et al., 2022). Theoretically, introducing a biocompatible network, formed by chemical molecules or proteins, might help avoid recurring problems by trapping proteins in the center layer of vitreous ice and thereby providing more evenly distributed particles (Figure 2C). Moreover, surface properties, such as the distribution of charge and hydrophobicity, play key roles in sample behavior in vitrification. Another potential strategy is burying the protein surface by putting the proteins in chemical cages. Chemical cages of different diameters are designed to fit proteins of distinct sizes (Figure 2D), and the proteins are enclosed in the cages by adding them during the self-assembly of the chemical cages under mild reaction conditions. The symmetry of these chemical cages offers at least two advantages: one, the carried proteins are randomly distributed in vitreous ice, which eliminates the occurrence of the preferred orientation problem, and two, effects on data processing are limited because the background noise introduced by the chemical cages could be averaged out due to the random distribution related to the density of the carrying protein.

Several chemical gels have already been applied in biological research, such as certain PEG hydrogels used for protein delivery and release (Kim and Cha, 2018; Sharma et al., 2019). Moreover, chemical cages are well studied in the fields of chemistry and synthetic biology (Zhukhovitskiy et al., 2016a; Zhukhovitskiy et al., 2016b; Wang et al., 2017). However, chemical cages are currently assembled under conditions (such as high temperature) that are not friendly to most biological macromolecules. If these hurdles can be overcome in future studies, the application of these networks and chemical cages to cryo-sample preparation will attract increased interest and have the potential to serve as a universal approach.

Lastly, an evaluation system that considers various factors, such as cost, applicability, flexibility, operation complexity, and sample solution requirements, could be established to assess the advantages and disadvantages of distinct modification methods through direct comparison and thus help the users make appropriate choices. We believe that this will benefit several researchers and strongly promote improvements in cryo-EM sample preparation. Given the increasing effort being devoted to the development of new technologies to mitigate recurring problems in the cryo-specimen preparation step, we expect an increase in the number of available methods, providing either alternative approaches or a universal approach, to address common obstacles encountered in cryo-sample preparation for high-resolution structure determination.
